# P-2183. Collocating HCV Treatment in a Medically Assisted Therapy Clinic in Dar es Salaam, Tanzania

**DOI:** 10.1093/ofid/ofae631.2337

**Published:** 2025-01-29

**Authors:** Christina Mmasa, Shyam Kottilil, Poonam Mathur, John Christian Rwegasha, Abubakar M Maghimbi, Joan J Rugemalila, Frank Aloyce Masao, Brenna M Roth, Iddi Haruna

**Affiliations:** Center for International Health, Education and Biosecurity-Tanzania (CIHEB-Tanzania), Dar es salaam, Dar es Salaam, Tanzania; Institute for Human Virology (IHV), University of Maryland School of Medicine, Baltimore, Maryland; University of Maryland, Baltimore, Maryland; Muhimbili National Hospital, Dar Es Salaam, Dar es Salaam, Tanzania; Center for International Health,Education & Biosecurity Tanzania, Dar es Salaam, Dar es Salaam, Tanzania; Muhimbili National Hospital, Dar Es Salaam, Dar es Salaam, Tanzania; Muhimbili National Hospital, Dar Es Salaam, Dar es Salaam, Tanzania; Henry M. Jackson Foundation for the Advancement of Military Medicine, Baltimore, Maryland; MUHIMBILI NATIONAL HOSPITAL, Dar es Salaam, Dar es Salaam, Tanzania

## Abstract

**Background:**

Tanzania’s estimated HCV prevalence is the highest in Africa at 2.7%. Among people with opioid use disorder (OUD) in Dar es Salaam, Tanzania’s most populous city, positive HCV Ab prevalence is estimated to be 16.2% to 50.2%. With limited HCV treatment available, patient-centered service delivery through collocating HCV treatment with other services, task shifting to non-specialists, and streamlined testing and treatment algorithms are needed to lower barriers to HCV treatment. The objective of this study was to determine the effectiveness of collocating HCV treatment in routine medically assisted therapy (MAT) clinic services to cure HCV infection in clinic attendees with OUD.

**Figure 1:**
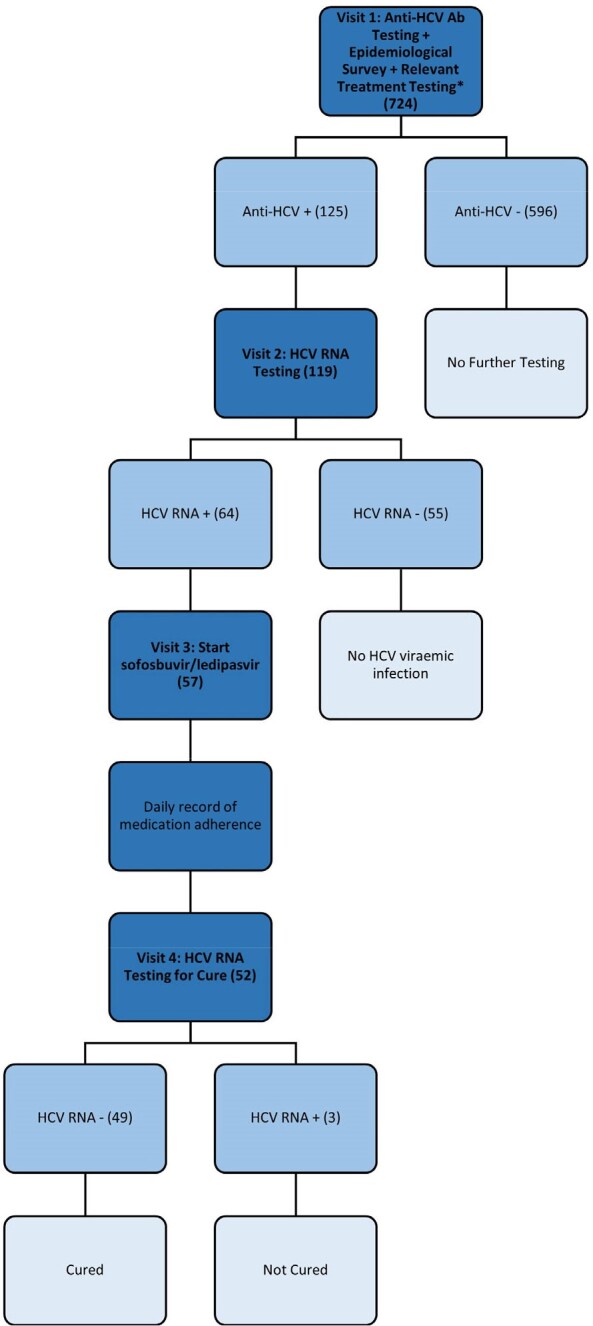
HCV testing and treatment algorithm completed by participants

**Methods:**

From December 2021 to July 2022, we enrolled regular attendees of the MAT clinic in Muhimbili National Hospital (MNH) (Figure 1) who underwent HCV Ab screening. Those with a positive HCV Ab were tested for HCV RNA and offered treatment with ledipasvir/sofosbuvir if this was detectable. Treatment was directly observed therapy, dispensed daily when the participant attended the clinic. Participants were tested for sustained virologic response (SVR), defined as undetectable HCV RNA at 12 weeks following treatment completion. **Figure 2.** Study participant demographics

**Figure:**
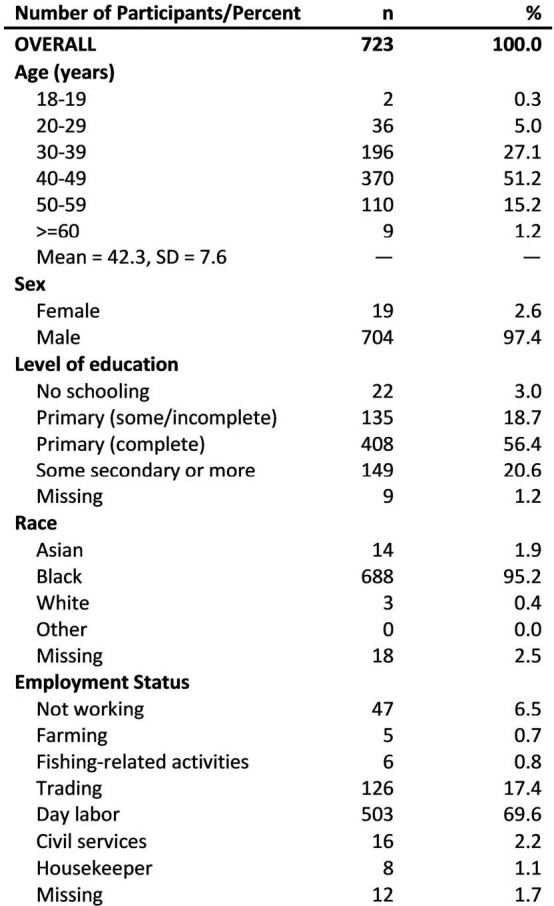
*Creatinine, Alanine aminotransferase

**Results:**

The study enrolled 724 regular attendees of the MAT clinic, most men (704/723, 97.37%) aged 40-49 (370/680, 51.2%) (Figure 2). Of those enrolled, 721/724 (99.6%) were screened for HCV, 125/721 (17.34%) had a positive HCV Ab, 119/125 (95.2%) underwent HCV RNA testing, 64/119 (53.8%) had detectable HCV RNA, 57/64 (89.1%) started treatment, 56/57 (98.2%) completed treatment, 52/56 (92.9%) were tested for SVR, and 49/52 (94.2%) achieved SVR at completion of the follow-up period, with an overall cure of 49/64 (76.6%) (Figure 3).

**Figure 3:**
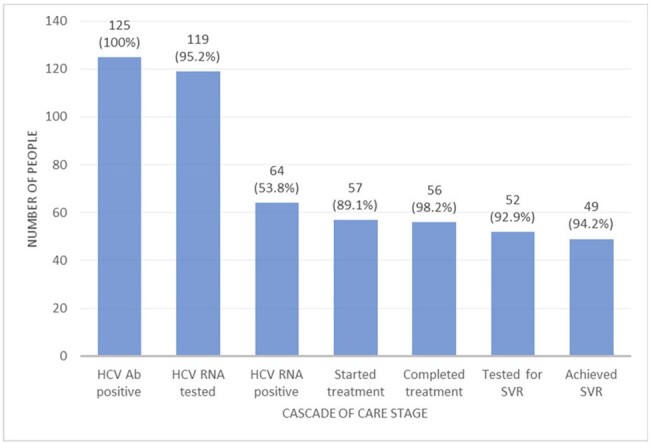
HCV care and treatment cascade

**Conclusion:**

In this study, 77% of participants with a detectable HCV RNA completed the treatment cascade and achieved SVR. Collaboration with medical staff and patient advocates to adapt the care and treatment model to the MAT clinic was important for success. Study visits and lab work were minimized to lower cost and barriers to adherence. Cost analyses and implementation studies should be done to evaluate the HCV care and treatment cascade in limited resource settings to build evidence for the benefits of these services and bolster government support.

**Disclosures:**

Shyam Kottilil, MD, PhD, Orsobio: Advisor/Consultant|Red Queen: Advisor/Consultant

